# Clinical significance of three-dimensional skeleton-arterial model in the management of delayed reconstruction of acetabular fractures

**DOI:** 10.1186/s12893-018-0362-y

**Published:** 2018-05-29

**Authors:** Xi Zhou, Qiang Zhang, Wenhao Song, Dongsheng Zhou, Yu He

**Affiliations:** 1 Department of Orthopaedics, Peking Union Medical College Hospital, Chinese Academy of Medical Sciences & Peking Union Medical College, No.1 Shuaifuyuan Wangfujing, Dongcheng District, Beijing, China; 2 Department of Orthopedics, Beijing Ditan Hospital, Capital Medical University, No.8 Jingshun East Street, Beijing, China; 3 Department of Orthopedic Surgery, Shandong Provincial Hospital Affiliated to Shandong University, 324 Jingwu Road, Ji’nan, Shandong China

**Keywords:** Rapid prototyping technique, Skeleton-arterial model, Acetabular fractures, Delayed reconstruction, Preoperative plan

## Abstract

**Background:**

Delayed reconstruction of acetabular fractures remains a challenging task for orthopedists because of malunion, fracture line absorption, and scar formation. Accurate osteotomy, interfragmentary release, and proper adaptation of plates are keys to successful surgery. Prevention of superior gluteal artery (SGA) injury induced by cleaning of the osteotylus and reduction of the sciatic notch is also important. Therefore, sufficient preoperative planning is essential. However, traditional planning methods do not readily provide direct visual and tactile feedback to surgeons. Rapid prototyping (RP) models have provided new opportunities in the preoperative planning of delayed reconstruction of acetabular fractures. We hypothesized that a three-dimensional (3D) skeleton-arterial model would improve both preoperative planning in the management of fractures and arteries and intraoperative assistance during delayed reconstruction of complex acetabular fractures.

**Methods:**

Eight patients were enrolled in this study. Data on the skeleton and arteries were obtained from computed tomography and angiography scans and used to produce RP models. Preoperative surgical planning and intraoperative assistance were performed using these models as references.

**Results:**

All 3D skeleton-arterial models were extremely accurate. Reduction and fixation were performed programmatically and smoothly, and management of the SGA was reliably executed according to a thorough preoperative plan. The mean surgical time and intraoperative blood loss were 224.4 min and 1250 ml, respectively. Among the eight patients, four underwent anatomic reduction and five had excellent functional outcomes at the final follow-up. No significant complications occurred.

**Conclusions:**

This 3D skeleton-arterial model is helpful for orthopedists in preoperative planning and intraoperative assistance.

## Background

Treatment of acetabular fractures remains one of most challenging tasks for orthopedists because of the complex anatomy, limited surgical access to fracture regions, and postoperative uncertainties [1, 2]. More problems remain to be solved for delayed reconstruction of acetabular fractures. It is more difficult to recreate the normal anatomy because of malunion, fracture line absorption, and scar formation between the fragments [3, 4]. The ultimate goal in the treatment of this injury is to restore hip function by reestablishing the normal anatomical structure [5, 6].

Accurate osteotomy and interfragmentary release in patients with severe malunion is usually achieved by surgeons with substantial experience and with the use of preoperative two-dimensional (2D) imaging [7]. Traditional planning methods do not readily provide direct visual and tactile feedback to surgeons. Proper adaptation of reconstruction plates is important for successful recreation of the normal anatomical structure. Nevertheless, intraoperative adaptation of a reconstruction plate is accomplished by a trial-and-error method. The risk of implant fatigue and cracking may be increased by repetitive bending [8]. Moreover, extra care is required for soft tissue management when injury to the superior gluteal artery (SGA) secondary to bone fragments or a surrounding callus is suspected. Damage to the SGA secondary to cleaning of the scar/osteotylus and reduction of the sciatic notch may have serious consequences [9–12]. These complex procedures are also associated with an increased surgical time and greater intraoperative blood loss. Therefore, proper morphometric evaluation and sufficient preoperative planning are essential before delayed reconstruction of acetabular fractures is begun.

Rapid prototyping (RP), also known as three-dimensional (3D) printing, is widely used in the engineering field and can be used to accurately reconstruct 3D objects from digital data. Medical RP techniques in clinical practice have been used for preoperative planning and rehearsal, intraoperative assistance, and educational tools [7, 13, 14]. The application of acetabular fracture biomodeling has been described by many researchers. Bagaria et al. [15] created a 3D model of an acetabular fracture. The preoperative planning for this fracture involved surgical simulation, template design, sizing and alignment of the implant, and production of the templates. Niikura et al. [16] used RP models to obtain informed consent for surgery in patients with complex acetabular fractures and educate patients and their families. Zeng et al. [17] used RP technology and computer-assisted virtual surgical procedures for preoperative planning in the treatment of acetabular fractures. These studies show that more accurate preoperative planning using RP models will reduce the operation time and significantly improve the outcome of acetabular fracture repair.

Although RP models have provided new opportunities in the preoperative planning of acetabular fracture surgery, the application of RP models to delayed reconstruction has not been described. Furthermore, how to protect the SGA during cleaning of the scar/osteotylus and reduction of the sciatic notch, which is a key point of surgery, has not been studied. The present study discusses the feasibility of preoperative planning in the management of fractures and arteries, intraoperative assistance, and communication with patients undergoing delayed reconstruction of complex acetabular fractures using 3D skeleton-arterial models.

## Methods

### Patients and imaging data

This study was approved by the Ethics Committee of Provincial Hospital Affiliated to Shandong University (No.2015028). Authors obtained written informed consent from all patients to participate this study. Patients with complex acetabular fractures treated with delayed reconstruction by internal fixation at our institution were included. All patients received standard preoperative and postoperative evaluations.

Eight patients were suspected to have arterial injury secondary to fracture fragments from September 2013 to March 2015. To obtain a definitive diagnosis, all eight patients were examined with computed tomographic angiography (CTA). A summary of the patients’ general demographics is shown in Table 1. The main reason for delayed surgery of acetabular fractures was mortal associated injuries, such as chest, abdominal, and craniocerebral injuries. Under these circumstances, the first priority is saving lives instead of fractures treatment. The minimum postoperative follow-up period was 18 months (mean, 26.1 months; range, 18–32 months).

**Table 1:** The detailed characteristics of patients

Case	Age/gender	Classification	Associated Injuries	Days following injury (day)	Surgical approach	Surgical time (min)	Intraoperative blood loss (ml)	Matta’s method	d’Aubigné–Postel score
1	40–49/1	Both columns	Chest and Craniocerebral injuries	67	Pararectus and Kocher–langenbeck approach	420	2300	Imperfect	Good
2	30–39/1	Both columns	Chest injuries	45	Ilio-inguinal and Kocher–langenbeck approach	285	1200	Anatomic	Excellent
3	20–29/1	Transverse and posterior wall	Abdominal injuries, closed internal degloving injury	42	Kocher–langenbeck approach	125	600	Imperfect	Excellent
4	30–39/1	T shape	Craniocerebral and abdominal injuries	36	Ilio-inguinal and Kocher–langenbeck approach	160	1000	Anatomic	Excellent
5	40–49/1	Both columns	Chest injuries	64	Ilio-inguinal and Kocher–langenbeck approach	310	1500	Imperfect	Good
6	30–39/2	Both columns	Abdominal injuries	54	Ilio-inguinal and Kocher–langenbeck approach	210	1900	Imperfect	Good
7	40–49/1	Both columns	Craniocerebral and chest injuries	29	Ilio-inguinal approach	150	800	Anatomic	Excellent
8	40–49/1	Both columns	Craniocerebral injuries	32	Pararectus approach	135	700	Anatomic	Excellent

**Fig. 1:**
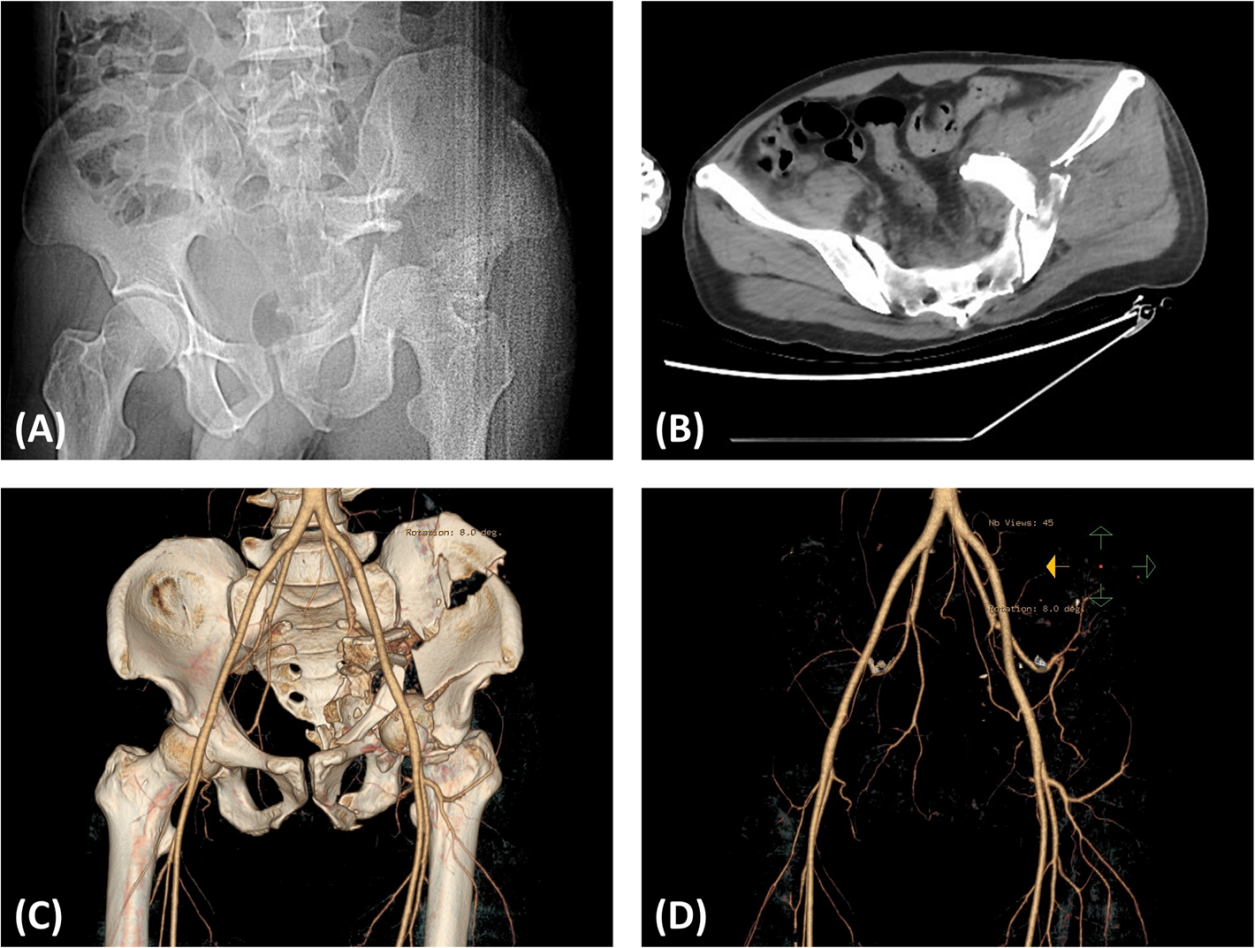
A patient (age between 40 and 50) sustained a fracture of his left acetabulum after a fall. Due to thoracic and head trauma, the surgery was performed 67 days later when the patient was in stable general condition. **a** Preoperative anteroposterior radiographs and (**b**) computed tomography scans of the pelvis were performed. Ruptured and rotated fragments of the sciatic notch were suspected to be causing injury to the superior gluteal artery. **c** To obtain a clear diagnosis, the patient was examined with computed tomography angiography. **d** No obvious arterial damage was found on computed tomography angiography; however, geometric information regarding fractures and arteries cannot be obtained from two-dimensional images

The data on the acetabular fractures were obtained from the patients’ computed tomography scans (Lightspeed VCT; GE, Fairfield, CT), and data on the arteries were obtained from CTA scans (Fig. 1). All data were saved as DICOM files.

### Rapid prototyping model

**Fig. 2:**
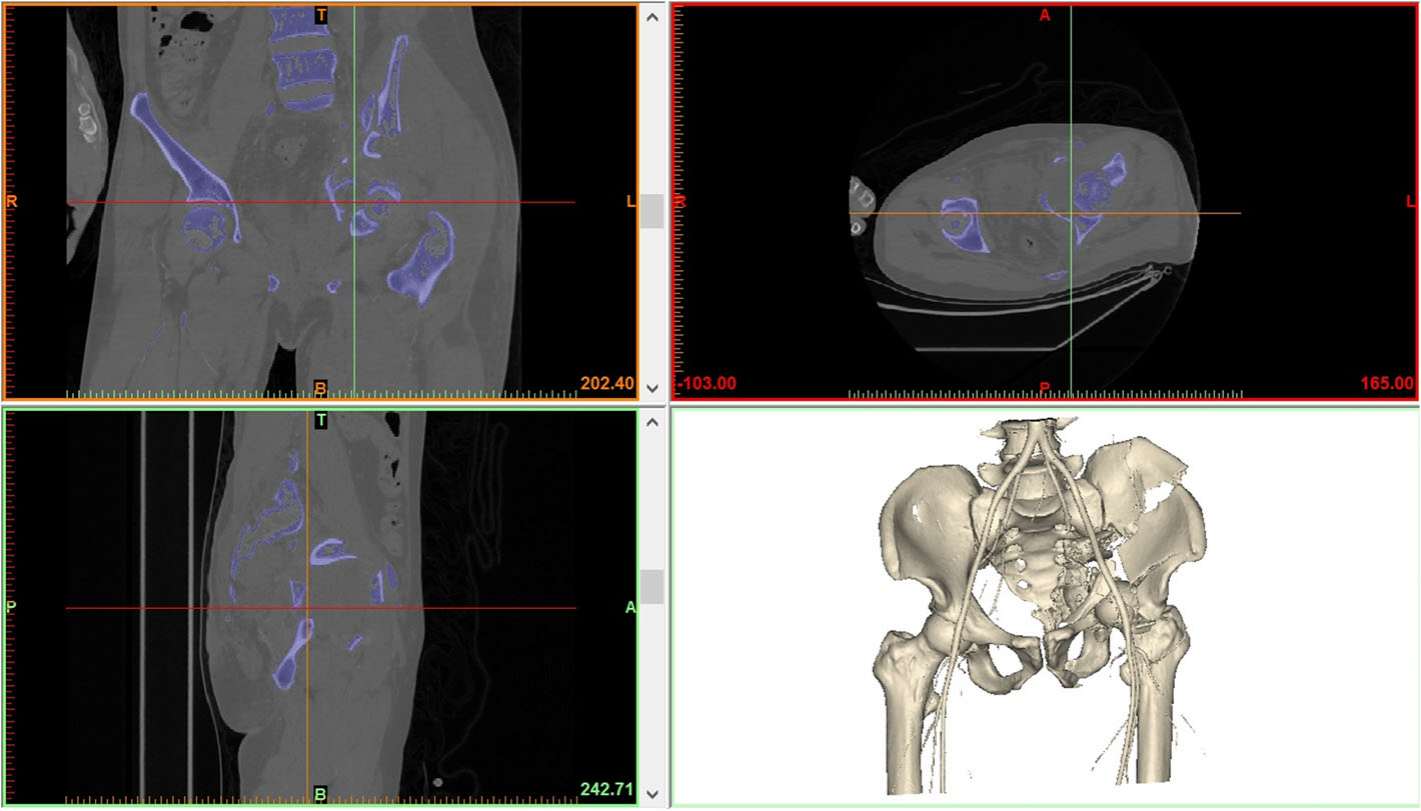
The two-dimensional images and three-dimensional reconstruction data were presented on the user interface of Mimics v15 software

**Fig. 3:**
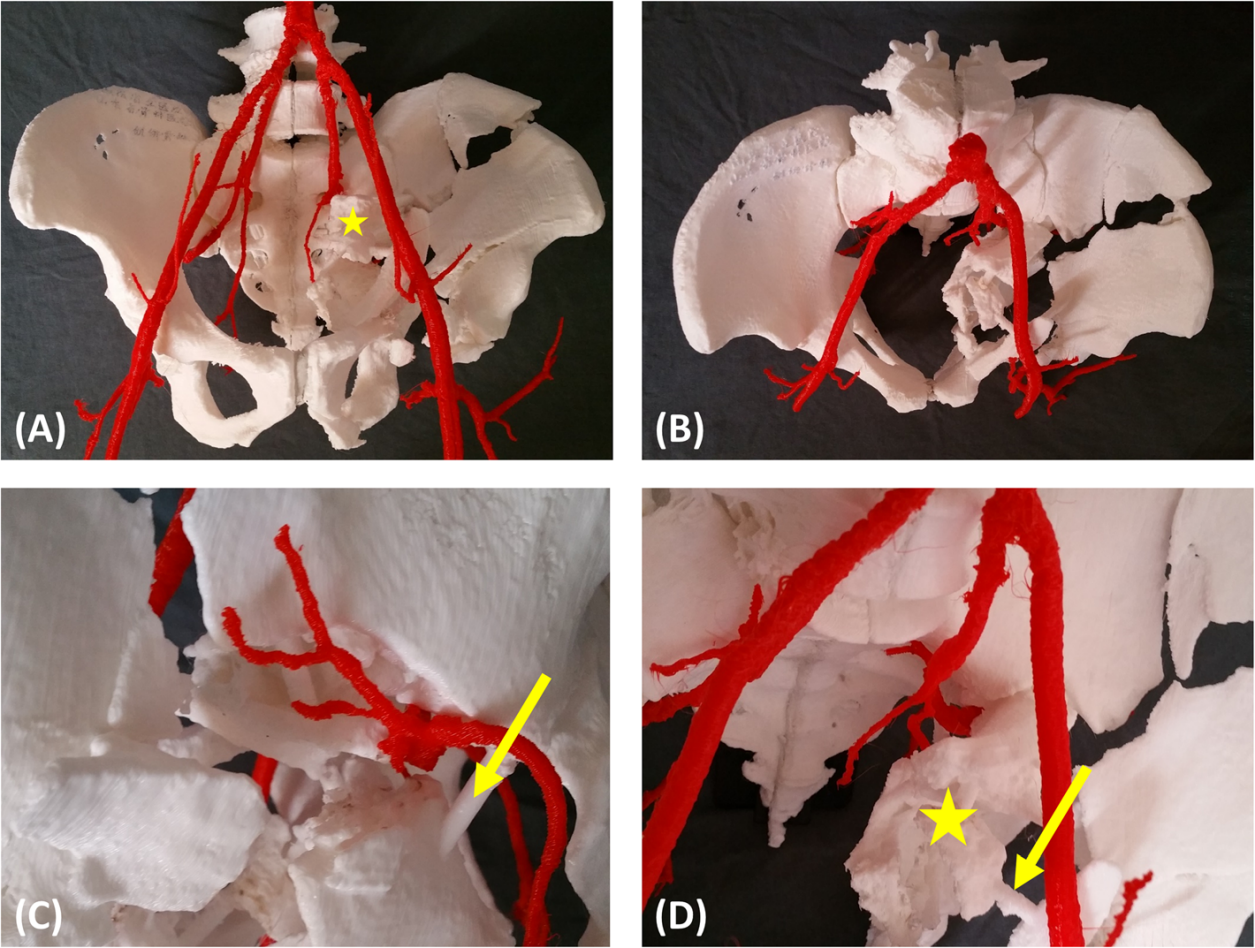
The details of the fractures were shown in the three-dimensional skeleton-arterial model. **a**, **b** The fracture characteristics and skeleton-arterial spatial relationships could be evaluated through different angles. The morphological observation, classification, and surgical approach were developed. **c**, **d** The superior gluteal artery was surrounded by several fracture fragments, and the sciatic notch was ruptured and rotated. Reduction of the sciatic notch was the key point in the surgical procedure (yellow star). However, simultaneous reduction of the sciatic notch and protection of the superior gluteal artery was not possible. We could not find a way to protect this artery in the preoperative plan; therefore, the need for ligation was determined intraoperatively. The free fragments were connected to the pelvis with a connecting rod to avoid changes in the spatial position (yellow arrow)

The skeletal and arterial data were imported into Mimics v15 software (Materialise, Leuven, Belgium). The software compiled the 2D data into 3D reconstruction data (Fig. 2), which were saved as STL files. The in-house RP machine (SRP400B; Waston Medical, Changzhou, China) of our department read the STL files and started the process of building the 3D skeleton-arterial model. If time and economic conditions allow, we can make two models: one for preoperative planning and the other for intraoperative assistance. The free fragments were connected to the pelvis with a connecting rod to prevent positional changes of the free fragments (Fig. 3).

We completed the whole production process after a simple training session. Without the influence of commercial factors, the manufacturing time and cost were able to be dramatically decreased.

### Preoperative surgical planning

The preoperative planning involved the following sequential steps: morphological observation and classification, surgical approach, osteotomy position in cases of severe malunion, reduction sequence and scenario, artery protection, and implant placement (prebent plate, screw position, and orientation).

**Fig. 4:**
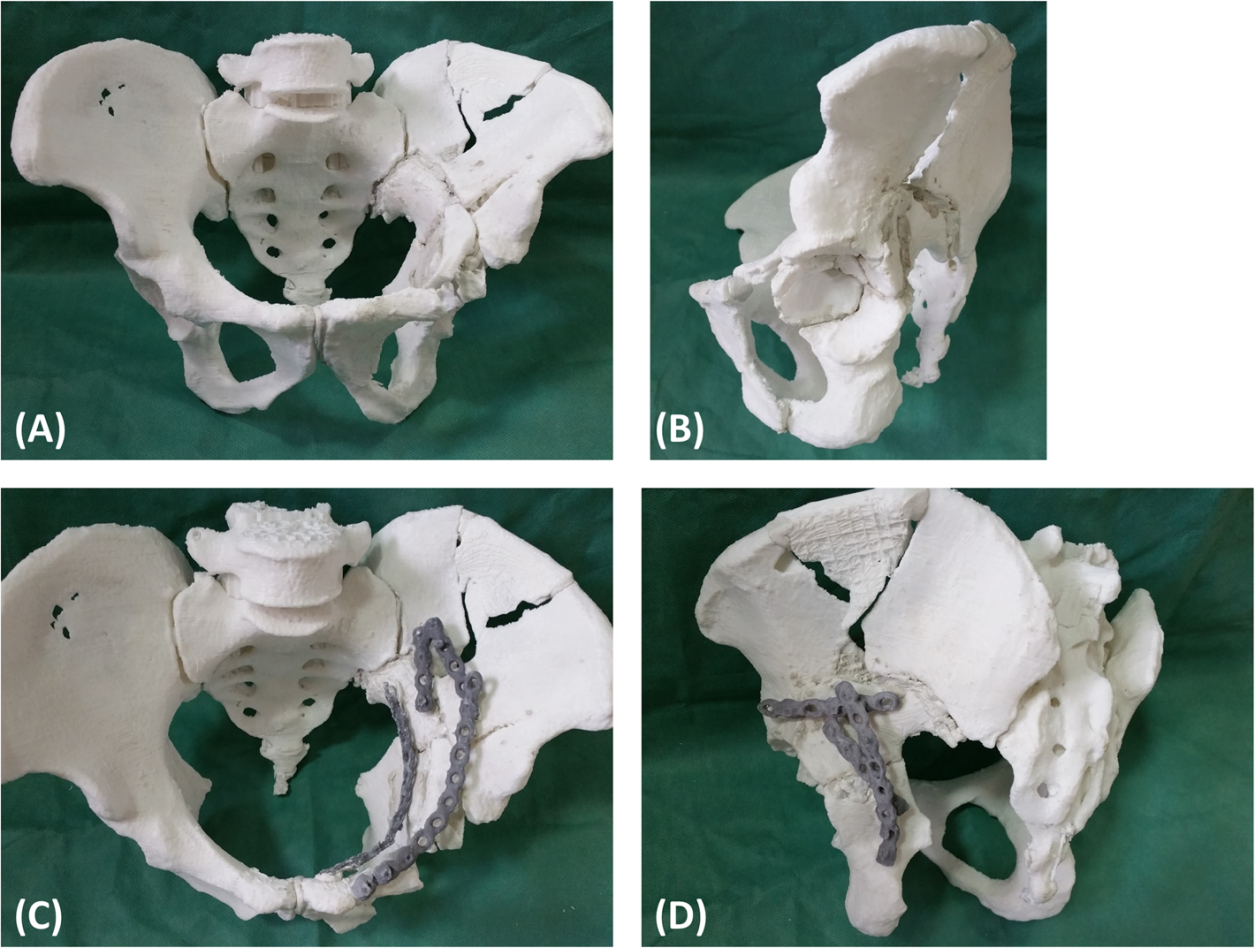
Delayed reconstruction of acetabular fractures was performed in the model. **a**, **b** Preoperative planning of the reduction sequence and scenario was performed, and (**c**, **d**) the implant placements were determined. The fracture of the iliac wing was not fixed because it was an old fracture with callus formation and stable fracture fragments

With a well-reduced acetabulum as a template, the pre-contour plate, screw position, and orientation were determined (Fig. 4). If reduction was not satisfactory, the uninjured acetabulum was used to assist. After development of the preoperative scenario, whether the SGA could be protected became clear. If this artery was difficult to protect, it was ligated to prevent uncontrolled bleeding during the operation.

### Intraoperative assistance

**Fig. 5:**
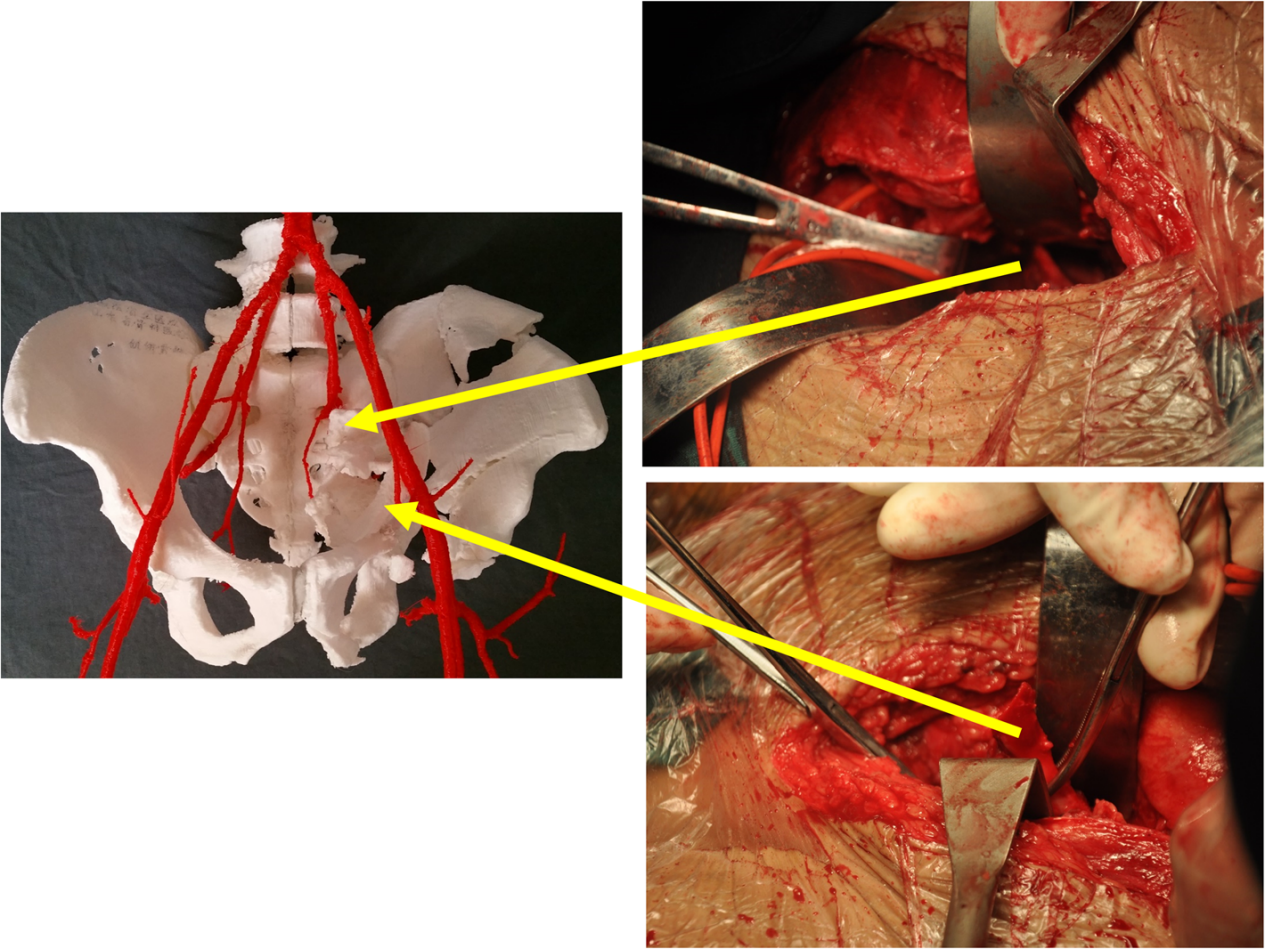
The models were applied to identify the operative landmarks and spatial relationships between the fracture fragments and arteries on the operating table

**Fig. 6:**
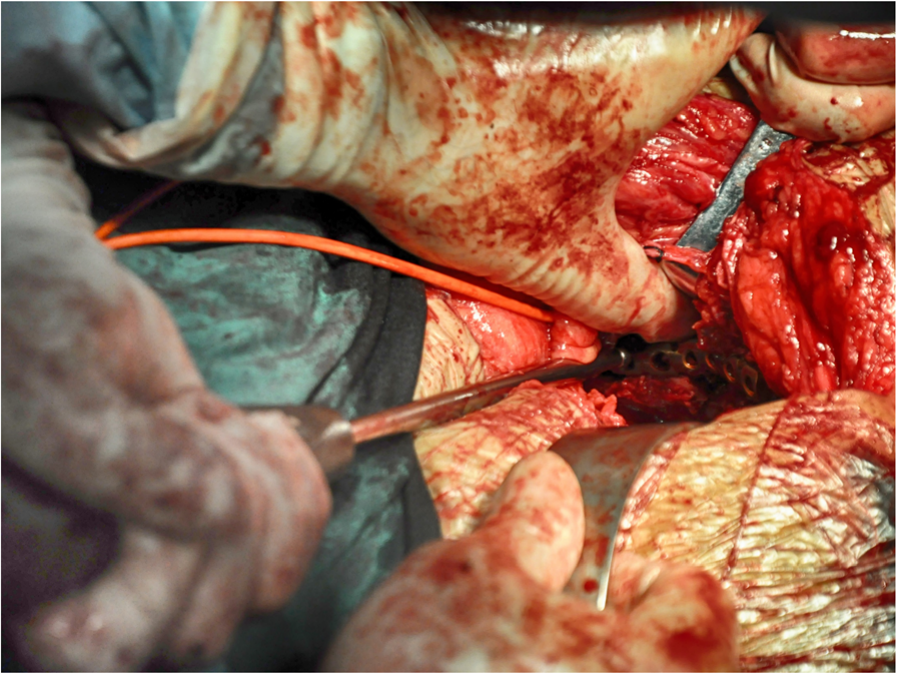
The indirect reduction technique and fixation were used with a pre-contoured construction plate during the operation

Anatomic landmarks were difficult to identify during the operation because of malunion, absorption of the fracture line, and osteotylus formation. The models were applied to identify the landmarks and spatial relationships of fracture fragments and arteries on the operating table (Fig. 5). Recognition of the bony landmarks helped surgeons with operation field exposure, reduction, fixation, and protection of the arteries. The pre-contoured plates were sterilized and applied in surgery. In addition, the indirect reduction technique could be used with the pre-contoured construction plate during the operation (Fig. 6).

### Assessment

The surgical time, intraoperative blood loss, reduction quality (Matta’s method [18]), and functional evaluation (Merle d’Aubigné–Postel score [19], Table 2) were recorded and analyzed to determine the operation effect. The reduction quality of acetabular fractures (Matta’s method) was graded as anatomical (0 to 1 mm of residual displacement), imperfect (1 to 3 mm) or poor (> 3 mm). The functional evaluation was based on the system of Merle d’Aubigné–Postel and was determined by adding the points: excellent = 18, good = 15 to 17, fair = 13 to 14, and poor < 13. The consistency of fixation management and the level of SGA injury between the planned and actual operations were recorded and analyzed to determine the reliability of preoperative planning with the 3D skeleton-arterial model. The anteroposterior radiographs of the pelvis were performed to evaluate fracture healing, heterotopic ossification, secondary dislocation, traumatic arthritis, and other complications at follow-up. CT examination can observe more details about fracture healing. In addition, the cost and time of production were recorded to evaluate the operability of the RP technique.

**Table 2:** System of Merle d’Aubigné–Postel

Pain	Score	Ability to walk	Score	Mobility	Score
No pain	6	Normal	6	Flexion of > 90°; abduction to 30°	6
Pain is mild and inconstant; normal activity	5	Without cane but with slight limp	5	Flexion between 80 and 90°; abduction of ≥15°	5
Pain is mild when walking; it disappears with rest	4	A long time with cane; short time without cane and with limp	4	Flexion between 60 and 80°; patient can reach his foot	4
Pain is tolerable with limited activity	3	With one cane, < 1 h; very difficult without a cane	3	Flexion between 40 and 60°	3
Pain is severe when walking; prevents any activity	2	Only with canes	2	Flexion under 40°	2
Pain is severe even at night	1	Only with crutches	1	No movement; pain or slight deformity	1
Pain is intense and permanent	0	None	0	Ankylosis with bad position of the hip	0

The clinical grade was based on a modification of the system of Merle d’Aubigné–Postel and was determined by adding the points: excellent = 18, good = 15 to 17, fair = 13 or 14, and poor = < 13

## Results

The detailed characteristics of all patients are shown in Tables 1 and 3. All 3D skeleton-arterial models used as a reference were found to be extremely accurate. There were six cases of both-column fractures, one case of transverse and posterior wall fractures, and one case of a T-shape fracture. Using the 3D skeleton-arterial model, the fracture morphology and arterial trend could be clearly observed without obscuration of the femoral head (Fig. 3). Fracture morphology was one of the factors used in selection of the surgical approach, which is especially significant in acetabular surgery. The ilio-inguinal and Kocher–Langenbeck approach were the most commonly used surgical approaches. The pararectus approach was a good choice for quadrilateral plate fractures. During surgery, accurate osteotomy and reduction tactics was precisely preformed with the help of the model (Fig. 4).

**Table 3:** Summary of preoperative and intraoperative management

Case	Fixation		Superior gluteal artery		Manufacturing Time (h/model)	Cost (dollar/model)
	Planned	Actual Operation	Planned	Actual Operation		
1	(anterior)	(anterior)	Ligate	Identical	42	37
	A 4-hole plate with 3 screws	Identical				
	A 4-hole plate with 2 screws					
	A 11-hole plate with 4 screws					
	A 11-hole plate with 4 screws					
	(Posterior)	(Posterior)				
	A 8-hole plate with 4 screws	A 8-hole plate with 4 screws				
	A 6-hole plate with 3 screws	A 6-hole plate with 3 screws				
	A 5-hole plate with 4 screws	A 5-hole plate with 4 screws				
		1 screw				
2	(anterior)	(anterior)	Reserve	Identical	52	45
	A 6-hole plate with 4 screws	Identical				
	A 14-hole plate with 4 screws					
	1 screw					
	(Posterior)	(Posterior)				
	A 4-hole plate with 3 screws	Identical				
	A 7-hole plate with 3 screws					
3	(Posterior)	(Posterior)	Reserve	Identical	43	37
	A 6-hole plate with 5 screws	Identical				
	A 8-hole plate with 5 screws					
	2 screws					
4	(anterior)	(anterior)				
	A 12-hole plate with 7 screws	Identical				
	(Posterior)	(Posterior)				
	A 8-hole plate with 5 screws	Identical				
	A 6-hole plate with 4 screws					
5	(anterior)	(anterior)	Reserve	Identical	45	40
	A 15-hole plate with 7 screws	Identical				
	A 4-hole plate with 4 screws					
	1 screw					
	(Posterior)	(Posterior)				
	A 6-hole plate with 4 screws	Identical				
	A 8-hole plate with 4 screws					
	1 screw					
6	(anterior)	(anterior)	Reserve	Identical	42	37
	A 15-hole plate with 8 screws	Identical				
	(Posterior)	(Posterior)				
	A 6-hole plate with 4 screws	Identical				
	A 8-hole plate with 4 screws					
7	(anterior)	(anterior)	Reserve	Identical	46	40
	A 12-hole plate with 6 screws	A 12-hole plate with 6 screws				
	A 4-hole plate with 3 screws	A 4-hole plate with 3 screws				
	A 4-hole plate with 4 screws	A 3-hole plate with 3 screws				
	A 5-hole plate with 4 screws	A 5-hole plate with 4 screws				
8	(anterior)	(anterior)	Reserve	Identical	50	45
	A 12-hole plate with 7 screws	Identical				
	A 9-hole plate with 4 screws					

**Fig. 7:**
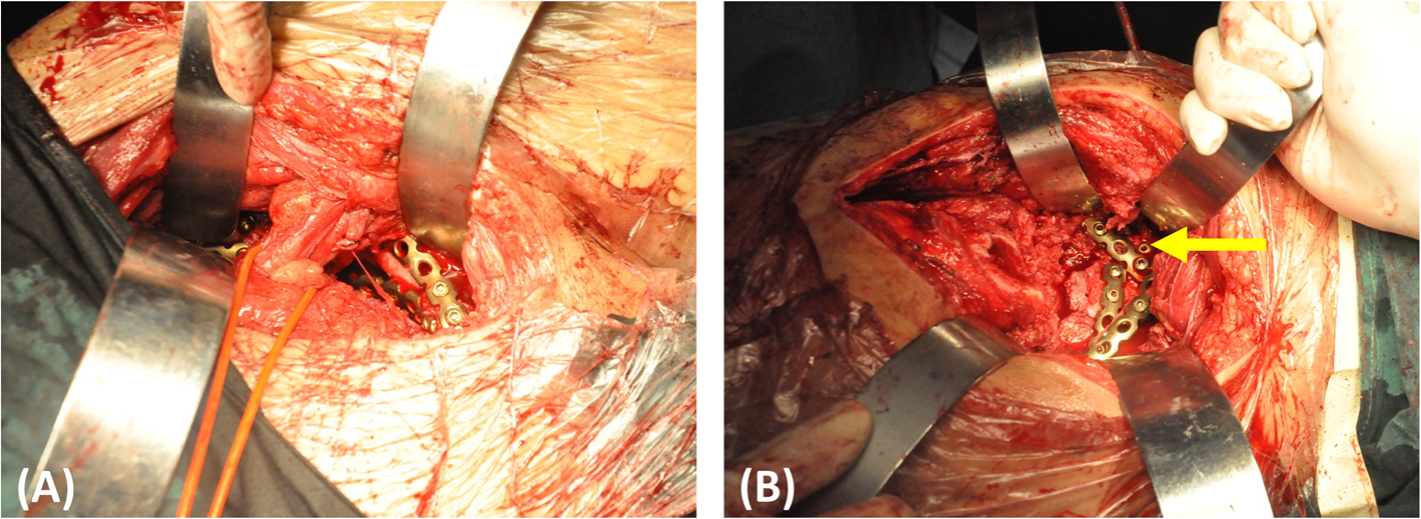
The pre-contoured plates selected for the models had a customized shape that perfectly matched the reconstructed acetabulum. The anterior (**a**) and posterior (**b**) implants were placed without difficulty, and no adjustments were required. However, there was a slight difference between the actual fixation management and the preoperative plan. One fragment was unreliably fixed by the plates, so a screw was unexpectedly required to enhance the fixation (yellow arrow)

**Fig. 8:**
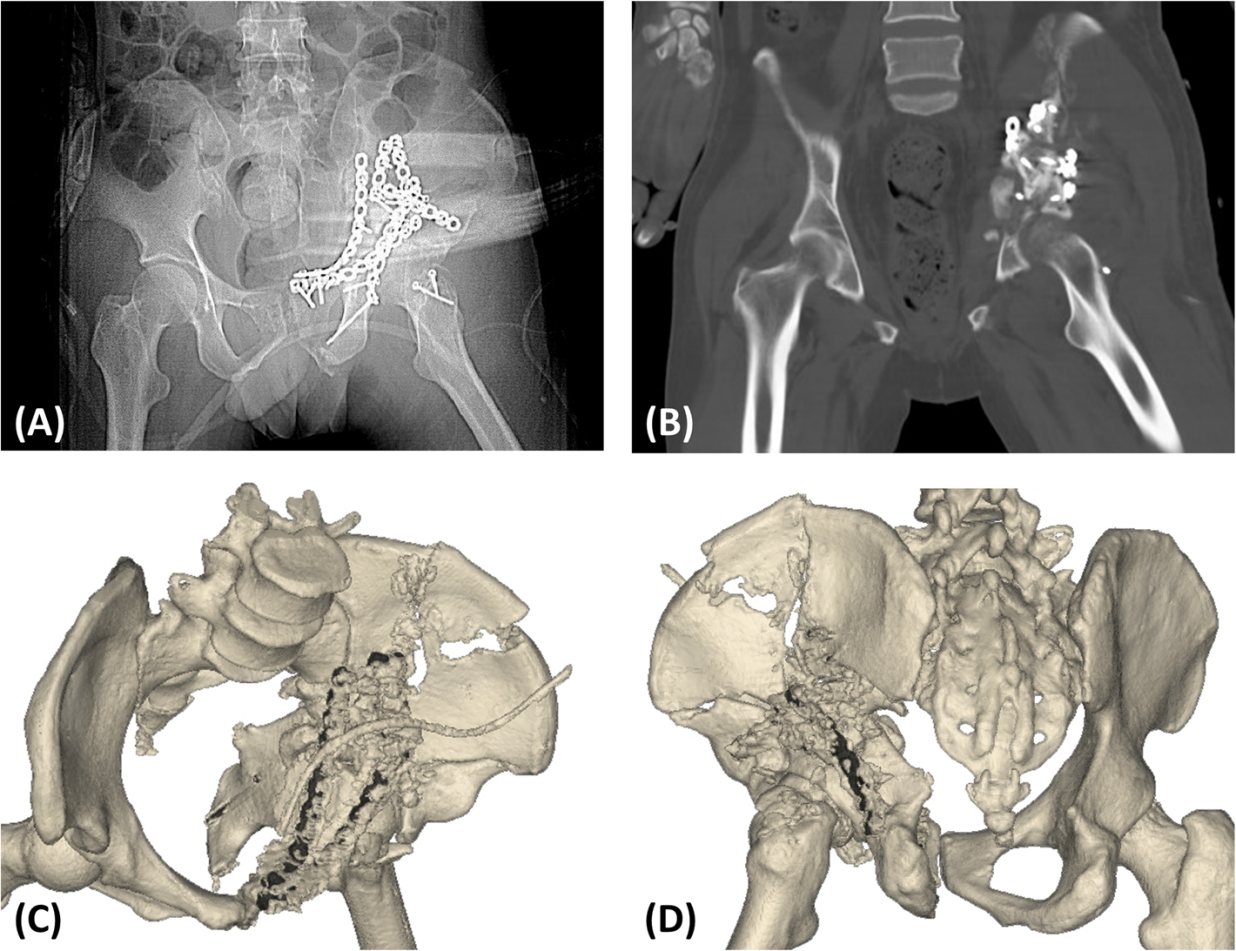
**a** The anteroposterior radiographs, **b** computed tomography scan, and (**c** and **d**) three-dimensional reconstruction of the postoperative pelvis were performed. The accuracy of reduction was imperfect. The orientation and length of all screws were suitable, and no articular penetration occurred

The reduction sequence and scenario of the fracture fragments and the key steps were simulated in the model. We tried every means possible to prevent SGA damage during reduction of the sciatic notch. In Case 1, we could not find a way to protect the SGA in the model, so ligation was determined to be necessary intraoperatively. In the other cases, the SGA was preserved by thorough preoperative planning. The appropriate implants were selected and pre-contoured. Application of the pre-contoured implants in the actual operation made the procedure easier to perform. The use of pre-contoured plates adjusted to the adequately reduced and patient-specific anatomy was found to be extremely helpful in guiding reduction (Figs. 7 and 8). The orientation and length of all screws were suitable, and no articular penetration occurred. In six cases, all implants were placed without difficulty, and no adjustments were needed. However, the actual fixation management was not completely identical to the preoperative plan in two cases. In Case 1, a fragment was unreliably fixed by plates, so a screw was unexpectedly needed to enhance the fixation. In Case 7, a four-hole pre-contoured construction plate could not be used because of limited space. To solve this problem, we cut a hole to shorten the length of the plate.

The procedures were performed by the same surgeon at the orthopedic department of our hospital. The mean surgical time and intraoperative blood loss were 224.4 min (range, 125–420 min) and 1250 ml (range, 600–2300 ml), respectively (Table 1). There were four cases of anatomic reduction and four cases of imperfect reduction. There were five cases of excellent functional outcomes and three cases of good functional outcomes at the final follow-up. No significant complications occurred.

The manufacturing time and cost of the 3D skeleton-arterial model were 45.7 h (range, 42–52 h) and 40.1 United States dollar (USD, range, 37–45 USD) (Table 3). The cost of model manufacturing was acceptable.

## Discussion

Acetabular fractures are serious intra-articular injuries caused by high energy [20]. Considering the complex anatomy, limited surgical access, and postoperative uncertainties, the treatment of acetabular fractures remains one of the most challenging tasks for orthopedists [1, 2]. Even more difficult problems are associated with delayed reconstruction of acetabular fractures. It is more difficult to recreate the normal structure in such cases because of malunion, fracture line absorption, and scar formation between the fragments [3, 4]. Early rehabilitation and excellent long-term functional outcomes, which are the ultimate treatment goals, can be established by anatomic reduction of the articular surface and stable fixation [5, 6]. Accurate osteotomy and interfragmentary release are usually performed in patients with severe malunion. Proper adaptation of reconstruction plates is important for successful recreation of the normal anatomical structure. Moreover, in patients with a suspected arterial injury, damage to the SGA secondary to cleaning of the scar/osteotylus and reduction of the sciatic notch may cause serious consequences. The surgical trauma and risk increase in association with these complex procedures. Unexpected bleeding, infection, neurovascular injury, or heterotopic ossification could make surgeons uncomfortable. Therefore, prior to beginning delayed reconstruction of acetabular fractures, proper morphometric evaluation and sufficient preoperative planning are essential. Traditionally, however, the preoperative plan was determined only by the doctor’s personal experience and preoperative 2D imaging results. This does not provide adequate visual and tactile feedback to surgeons.

The RP technique has a promising future in the orthopedics field [7, 13, 14]. The application of acetabular fracture biomodeling has been described by many researchers [15–17]. RP models offer many advantages over traditional methods, including preoperative planning and rehearsal, intraoperative assistance and educational tools, and good postoperative outcomes. However, the application of 3D skeleton-arterial model in the management of delayed reconstruction of acetabular fractures has not been reported. In this study, we focused on the design and support of preoperative planning using the RP model, not on performance of the operative technique.

This 3D skeleton-arterial model enabled surgeons to obtain clear and reliable virtual information. Observation of the fracture morphology without obscuration of the femoral head helped to classify the fracture and choose the optimal surgical approach. Knowledge of the precise spatial relationships and receiving tactile feedback allowed for more effective performance of the osteotomy, reduction, and fixation. Furthermore, the spatial relationships between the fragments and artery and the real-time feedback for the procedures could be evaluated during the virtual surgery. Therefore, the surgeon could determine whether the SGA could be preserved. The position of the SGA in the sciatic notch makes it prone to injury from fracture fragments and the reduction procedure [9–12]. In Case 1, the SGA was surrounded by several fracture fragments, and the sciatic notch was ruptured and rotated (Fig. 3). Simultaneous reduction of the sciatic notch and protection of the SGA simultaneous was not possible. We could not find a way to protect the artery in the model; therefore, the decision regarding ligation was made intraoperatively. In the other cases, the SGA was preserved by thorough preoperative planning.

Delayed reconstruction of acetabular fractures is a complicated procedure that is often performed to correct deformities after malunion. During these complicated procedures, a significant amount of time is spent contouring the implants. We reduced the operating time by pre-contouring the plates based on the RP models. In six cases, all implants were placed without difficulty, and no adjustments were needed. The pre-contoured plates selected based on the models had a customized shape that perfectly matched the reconstructed acetabulum. The orientation and length of all screws were suitable, and no articular penetration occurred. However, minor adjustments were required in two cases. The 3D models were based on the skeletal and arterial systems, and other soft tissues were neglected. The full biological characteristics of the fracture could not be reflected by these models. When problems are encountered during the operation, a revised management plan should be created in a timely manner. Changing the plan during surgery requires a high level of technical skill. We must keep in mind that the surgeon’s experience and skill are the most significant factors in delayed reconstruction of acetabular fractures. Therefore, the procedures should be performed by experienced and skilled surgeons.

Previous studies [3, 4] showed that the mean surgical time was 195–240 min and the mean blood loss was 1600 ml, which are slightly different than the current results (224 min and 1250 ml). However, there was no comparability among different case series due to diverse fracture characteristics and surgeons skills. At the final follow-up, four of eight patients achieved anatomic reduction and five of eight patients achieved excellent functional outcomes. Theoretically, the surgical trauma and risk will decrease and the outcomes of reduction and function will improve through use of a sophisticated preoperative plan. However, this was a small-sample study with some potentially confounding variables. Definite quantitative conclusions are difficult because of the limited number of patients. Based on our subjective feelings and past experience, our 3D skeleton-arterial model allowed surgery to be performed programmatically and smoothly.

The patients and their families obtained benefits from the models. An understanding of the fracture condition, surgical procedures, and operative risk would help patients and their families in making decisions. A benefit of using the noncommercial in-house 3D printer in our department is that the mean manufacturing time (45.7 h) and mean cost (40.1 USD) of the 3D skeleton-arterial model were decreased dramatically. The cost of model manufacturing was acceptable. A lower cost and shorter production time will allow for a wider range of applications.

There were limitations to the current study. Because of the limited number of patients in this small-sample study, it is difficult to make definite quantitative conclusions. This limitation may be resolved by randomized control trials with larger samples and long-term follow-up. The 3D skeleton-arterial models were created based on the skeletal and arterial systems without considering the effects of muscles and ligaments. The surgical plan must be modified when soft tissue impedes the operation. Furthermore, the properties of human tissue cannot be imitated by models. In this model, the arteries were represented by hard objects; this may have affected the preoperative plan. Therefore, there might be a necessary for a reconsidered management plan during the operation. However, this skeleton-arterial model still provides helpful clinical data for surgeons.

## Conclusion

This 3D skeleton-arterial model is helpful for orthopedists with respect to preoperative planning, intraoperative assistance, and communication with patients. Indirect benefits of a sophisticated preoperative plan are decreased trauma and surgical risk and improved reduction and function. This model is a powerful instrument with potential to be extremely useful in the preoperative planning of delayed reconstruction of acetabular fractures. Considering the complexity of the operation itself, however, the procedures should be performed by experienced and skilled surgeons.

## Abbreviations

2D: Two-dimensional
3D: Three-dimensional
CT: Computed tomography
CTA: Computed tomographic angiography
RP: Rapid prototyping
SGA: Superior gluteal artery
USD: United States dollar
